# Antituberculosis drug resistance patterns in adults with tuberculous meningitis: results of haydarpasa-iv study

**DOI:** 10.1186/s12941-015-0107-z

**Published:** 2015-11-04

**Authors:** Seniha Senbayrak, Nuri Ozkutuk, Hakan Erdem, Isik Somuncu Johansen, Rok Civljak, Ayse Seza Inal, Uner Kayabas, Ebru Kursun, Nazif Elaldi, Branislava Savic, Soline Simeon, Emel Yilmaz, Olga Dulovic, Derya Ozturk-Engin, Nurgul Ceran, Botond Lakatos, Oguz Resat Sipahi, Mustafa Sunbul, Mucahit Yemisen, Selma Alabay, Bojana Beovic, Aysegul Ulu-Kilic, Yasemin Cag, Melanie Catroux, Asuman Inan, Gorana Dragovac, Ozcan Deveci, Recep Tekin, Hanefi Cem Gul, Gonul Sengoz, Katell Andre, Arjan Harxhi, Yves Hansmann, Serkan Oncu, Sukran Kose, Oral Oncul, Emine Parlak, Alper Sener, Gulden Yilmaz, Umit Savasci, Haluk Vahaboglu

**Affiliations:** Department of Infectious Diseases and Clinical Microbiology, Haydarpasa Numune Training and Research Hospital, Istanbul, Turkey; Department of Medical Microbiology, Celal Bayar University School of Medicine, Manisa, Turkey; Department of Infectious Diseases and Clinical Microbiology, Gulhane Medical Academy, Etlik, Ankara Turkey; Department of Infectious Diseases Q, Odense University Hospital, Odense, Denmark; Department of Infectious Diseases, Dr. Fran Mihaljevic University Hospital for Infectious Diseases, University of Zagreb School of Medicine, Zagreb, Croatia; Department of Infectious Diseases and Clinical Microbiology, Cukurova University School of Medicine, Adana, Turkey; Department of Infectious Diseases and Clinical Microbiology, Inonu University School of Medicine, Malatya, Turkey; Department of Infectious Diseases and Clinical Microbiology, Baskent University School of Medicine, Adana, Turkey; Department of Infectious Diseases and Clinical Microbiology, Cumhuriyet University School of Medicine, Sivas, Turkey; National Reference Laboratory for Tuberculosis, Institute of Microbiology and Immunology, Faculty of Medicine, University of Belgrade, Belgrade, Serbia; Department of Infectious and Tropical Diseases, University Hospital of Pontchaillou, Rennes, France; Department of Infectious Diseases and Clinical Microbiology, Uludag University School of Medicine, Bursa, Turkey; Clinic for Infectious and Tropical Diseases, Clinical Centre of Serbia, Faculty of Medicine, University of Belgrade, Belgrade, Serbia; Department of Infectious Diseases, Saint Laszlo Hospital, Budapest, Hungary; Department of Infectious Diseases and Clinical Microbiology, Ege University School of Medicine, Izmir, Turkey; Department of Infectious Diseases and Clinical Microbiology, Ondokuz Mayis University School of Medicine, Samsun, Turkey; Department of Infectious Diseases and Clinical Microbiology, Istanbul University Cerrahpasa School of Medicine, Istanbul, Turkey; Department of Infectious Diseases and Clinical Microbiology, Erciyes University School of Medicine, Kayseri, Turkey; Department of Infectious Diseases, University Medical Centre, Ljubljana, Slovenia; Department of Infectious Diseases and Clinical Microbiology, Lutfi Kirdar Training and Research Hospital, Istanbul, Turkey; Department of Infectious Diseases, Poitiers University Hospital, Poitiers, France; IPH of Vojvodina, Department of Prevention and Control of Diseases, Medical Faculty, University of Novi Sad, Novi Sad, Serbia; Department of Infectious Diseases and Clinical Microbiology, Dicle University School of Medicine, Diyarbakir, Turkey; Department of Infectious Diseases and Clinical Microbiology, Haseki Training and Research Hospital, Istanbul, Turkey; Department of Infectious Diseases, Dax Hospital, Dax, France; Service of Infectious Disease, University Hospital Center of Tirana, Tirana, Albania; Department of Infectious Diseases, University Hospital, Strasbourg, France; Department of Infectious Diseases and Clinical Microbiology, Adnan Menderes University School of Medicine, Aydin, Turkey; Department of Infectious Diseases and Clinical Microbiology, Tepecik Training and Research Hospital, Izmir, Turkey; Department of Infectious Diseases and Clinical Microbiology, GATA Haydarpasa Training Hospital, Istanbul, Turkey; Department of Infectious Diseases and Clinical Microbiology, Ataturk University School of Medicine, Erzurum, Turkey; Department of Infectious Diseases and Clinical Microbiology, Onsekiz Mart University School of Medicine, Canakkale, Turkey; Department of Infectious Diseases and Clinical Microbiology, Ankara University School of Medicine, Ankara, Turkey; Department of Infectious Diseases and Clinical Microbiology, Goztepe Training and Research Hospital, Medeniyet University, Istanbul, Turkey

**Keywords:** Tuberculosis, Meningitis, Resistance, MDR, Isoniazid

## Abstract

**Background:**

Tuberculous meningitis (TBM) caused by *Mycobacterium tuberculosis* resistant to antituberculosis drugs is an increasingly common clinical problem. This study aimed to evaluate drug resistance profiles of TBM isolates in adult patients in nine European countries involving 32 centers to provide insight into the empiric treatment of TBM.

**Methods:**

*Mycobacterium tuberculosis* was cultured from the cerebrospinal fluid (CSF) of 142 patients and was tested for susceptibility to first-line antituberculosis drugs, streptomycin (SM), isoniazid (INH), rifampicin (RIF) and ethambutol (EMB).

**Results:**

Twenty of 142 isolates (14.1 %) were resistant to at least one antituberculosis drug, and five (3.5 %) were resistant to at least INH and RIF, [multidrug resistant (MDR)]. The resistance rate was 12, 4.9, 4.2 and 3.5 % for INH, SM, EMB and RIF, respectively. The monoresistance rate was 6.3, 1.4 and 0.7 % for INH, SM and EMB respectively. There was no monoresistance to RIF. The mortality rate was 23.8 % in fully susceptible cases while it was 33.3 % for those exhibiting monoresistance to INH, and 40 % in cases with MDR-TBM. In compared to patients without resistance to any first-line drug, the relative risk of death for INH-monoresistance and MDR-TBM was 1.60 (95 % CI, 0.38–6.82) and 2.14 (95 % CI, 0:34–13:42), respectively.

**Conclusion:**

INH-resistance and MDR rates seemed not to be worrisome in our study. However, considering their adverse effects on treatment, rapid detection of resistance to at least INH and RIF would be most beneficial for designing anti-TB therapy. Still, empiric TBM treatment should be started immediately without waiting the drug susceptibility testing.

## Background

Tuberculosis (TB) continues to be a serious and challenging global health problem. World health organization (WHO) estimated that 9 million people developed TB and 1.5 million people died of this disease in 2013. The same report emphasized the development of multidrug resistant tuberculosis [MDR-TB, defined as resistance to at least isoniazid (INH) and rifampicin (RIF)] in an estimated 480,000 people in 2013, of which only half of them could be diagnosed [1]. Extrapulmonary tuberculosis accounts for more than 15 % of all tuberculosis cases [1]. Tuberculous meningitis (TBM) is a very serious form of extrapulmonary TB, accounting for 5–10 % and requiring prompt treatment [2]. The mortality rate for TBM ranges between 20 and 69 % worldwide with up to half of survivors experiencing irreversible sequelae. One of the most important factors affecting the prognosis is early diagnosis and proper treatment [3].

The sensitivities of cerebrospinal fluid (CSF) microscopic examination and culture are low because of insufficient numbers of bacilli in the CSF. Moreover, prolonged periods of time for recovery lead to significant delays in obtaining the results of drug susceptibility tests (DSTs). However, initiation of effective anti-tuberculous treatment in the early stages of TBM is significantly associated with reduced rates of mortality and sequelae [4]. Thus, the diagnosis of TBM is often based on epidemiological, clinical and radiological findings, followed by standard empirical treatment without awaiting culture results [2, 4]. However, the effectiveness of the standard treatment is low in patients with TBM caused by drug-resistant strains. INH has the highest CSF penetration among first-line antituberculosis agents and even monoresistance to INH may adversely affect the treatment outcomes. Multidrug Resistant Tuberculous Meningitis (MDR-TBM) increases worldwide and the prognosis is even worse in patients with MDR-TBM [5, 6].

Reports on drug resistance rates in TBM are limited and often anecdotal due to difficulties in obtaining the results of DST [7, 8]. In this study, we evaluated drug resistance profiles of TBM in adult patients in nine European countries involving 32 centers in an attempt to provide more insight into the empiric treatment of TBM.

## Methods

Haydarpasa-IV is a retrospective, multinational, and multicenter study. The initial two analyses from the Haydarpasa database, the first recommending a diagnostic algorithm and the second one providing a new severity index, were published previously [3, 9]. A Microsoft Windows-based computer database was designed and data were collected from 32 centers in nine countries (Turkey, Slovenia, Serbia, Romania, Hungary, France, Denmark, Croatia and Albania). The study protocol was approved by the Institutional Review Board of Istanbul Fatih Sultan Mehmet Training and Research Hospital.

DST results of *M. tuberculosis* strains isolated from CSF samples of 142 adult patients [76 females (53.5 %); mean age 44.9 ± 19.8 years] with TBM between 2000 thorough 2012 were evaluated. The study group comprised of 124 new TBM cases (87.3 %) and 15 previously treated cases (10.6 %). In three patients, previous history was missing in the hospital records (2.1 %). The inclusion criteria were age older than 14 years, presence of at least one positive CSF culture result and culture-based DST results for the first-line drugs.

A total of 142 *M. tuberculosis* isolates recovered from CSF samples were tested by culture-based DSTs for susceptibility to four first-line drugs, streptomycin (SM), INH, RIF and ethambutol (EMB). Culture-based DSTs were performed using different methods in individual centers. Of 142 isolates, 116 (81.7 %) were tested using automated culture systems including BACTEC MGIT 960 (n = 94), BACTEC 460 (n = 9), BACTEC 9000 MB (n = 7) (Becton–Dickinson Diagnostic Systems, Sparks, MD, USA) and BacT/Alert MB (n = 6) (bioMérieux Diagnostics, Durham, NC, USA) in full compliance with the manufacturers’ recommendations. The remaining 26 isolates were tested by the solid culture proportion method on Lowenstein–Jensen medium (n = 22) and on Middlebrook 7H10 agar (n = 4) using the standard protocol.

Data were analyzed using the SPSS software package, version 16.0 (SPSS Inc, Chicago, IL, USA). We determined the unadjusted association between resistance (INH-monoresitance and MDR) and subsequent death by using the χ^2^ test and Fischer’s exact test, and P < 0.05 was accepted as significant. We calculated an odds ratio and a 95 % confidence interval to evaluate the strength of the association and the precision of the effect.

## Results

*Mycobacterium tuberculosis* strains isolated from 122 patients (85.9 %) were found to be susceptible to all four first-line drugs tested, the remaining 20 strains (14.1 %) were resistant to at least one anti-TB drug. The highest resistance rate was seen for INH with 17 strains (12 %), followed by SM (4.9 %, n = 7), EMB (4.2 %, n = 6), and RIF (3.5 %, n = 5). Monoresistance rate was also the highest for INH with nine strains (6.3 %), being 1.4 % (n = 2) and 0.7 % (n = 1) for SM and EMB, respectively. In the absence of monoresistance to RIF, all RIF-resistant cases were found to have MDR-TBM (Table 1). In this study, five of nine cases with INH-monoresistance were reported from Turkey, two from France, one from Denmark, and one from Romania.

**Table 1:** First-line drug resistance rates of *M. tuberculosis strains* isolated from cerebrospinal fluids

	n	%
*Total number of strains tested*	*142*	*100.0*
*Susceptible to all four drugs*	*122*	*85.9*
*Any resistance*	*20*	*14.1*
INH	17	12.0
RIF	5	3.5
EMB	6	4.2
SM	7	4.9
*Monoresistance*	*12*	*8.5*
INH	9	6.3
RIF	0	0.0
EMB	1	0.7
SM	2	1.4
*Multidrug Resistance (MDR)*	*5*	*3.5*
INH + RIF	1	0.7
INH + RIF + EMB	2	1.4
INH + RIF + EMB + SM	2	1.4
*Other patterns*	*3*	*2.1*
INH + SM	2	1.4
INH + EMB + SM	1	0.7

*SM* streptomycin, *INH* isoniazid, *RIF* rifampicin, *EMB* ethambutol

Characteristic features and comorbid conditions of tuberculous meningitis patients and the distribution of drug susceptibility according to the countries participating in the study are shown in Tables 2, 3 respectively.

**Table 2:** Characteristics and drug susceptibility patterns of tuberculous meningitis patients

Drug resistance	Age (median, range)	Gender (male) n (%)	Malignity n (%)	DM n (%)	CKD n (%)	IS n (%)	HIV n (%)	Dead n (%)
Fully sensitive (n: 122)	45.9 (17–87)	60 (49.2)	6 (4.9)	22 (18.0)	5 (4.1)	16 (13.1)	2 (1.6)	29 (23.8)
Monoresistance to INH (n: 9)	35.4 (19–89)	1 (11.1)	–	1 (11.1)	1 (11.1)	1 (11.1)	1 (11.1)	3 (33.3)
MDR (n: 5)	37.6 (19–70)	1 (20.0)	–	1 (20.0)	–	1 (20.0)	–	2 (40.0)
Other patterns (n: 6)	43.3 (28–81)	4 (16.7)	–	–	1 (16.7)	–	–	1 (16.7)
Total (n: 142)	44.9 (17–89)	66 (46.5)	6 (4.2)	24 (12.7)	7 (4.9)	18 (16.9)	3 (2.1)	35 (24.6)

*MDR* multidrug resistance, *INH* isoniazid, *DM* diabetes mellitus, *CKD* chronic kidney disease, *IS* immunosupression

**Table 3:** The distribution of drug susceptibility according to the countries participating in the study

Country	Number of strains tested	R n (%)	INH-monoresistance n (%)	MDR-TBM n (%)	Other patterns n (%)	Dead n (%)	Resistance of strains from patiens dead n (%)
Turkey	69	12 (17.4)	5 (7.2)	3 (4.3)	4 (5.8)	9 (13.3)	2 (22.2)
Denmark	25	1 (4.0)	1 (4.0)	–	–	8 (32.0)	1 (12.5)
Croatia	19	–	–	–	–	9 (47.4)	–
Serbia	11	2 (18.2)	–	2 (18.2)	–	5 (45.5)	1 (20.0)
France	9	4 (44.4)	2 (22.2)	–	2 (22.2)	3 (33.3)	2 (66.7)
Hungary	4	–	–	–	–	–	–
Slovenia	3	–	–	–	–	1 (33.3)	–
Albania	1	–	–	–	–	–	–
Romania	1	1 (100.0)	1 (100.0)	–	–	–	–
Total	142	20 (14.1)	9 (6.3)	5 (3.5)	6 (4.2)	35 (24.6)	6 (17.1)

*R* any resistance, *TBM* tuberculous meningitis, *MDR* multidrug resistance, *INH* isoniazid

Overall, MDR-TBM was identified in five patients (3.5 %), all of which were new cases (three from Turkey, two from Serbia). Among MDR-TBM cases, two patients were also resistant to EMB and two patients were also resistant to EMB and SM, while one patient was not resistant to any drugs tested other than RIF and INH. HIV status was available for only 128 cases. Of these, only three patients (2.3 %) were positive for HIV. On the other hand, all of the MDR-TBM cases were HIV-negative.

The overall mortality rate was 24.6 % with 35 cases. All patients, but one have died during treatment. The median time of death was 37 days (min: 1, max: 692). The average treatment duration in patients died were 4.4 months, and it was 12.7 months in the survivors.

Mortality was seen in 23.8 % (n = 29) of fully susceptible cases, in 30 % (n = 6) of those who were resistant to at least one anti-TB drug, in 33.3 % (n = 3) of cases who exhibited monoresistance to INH, and in 40 % (n = 2) of cases with MDR-TBM. In comparison with patients without resistance to any all first-line drugs, the relative risk of death for INH-monoresistance and MDR-TBM was 1.60 (95 % CI, 0.38–6.82) and 2.14 (95 % CI, 0:34–13:42), respectively. However, these differences were not statistically significant.

## Discussion

Tuberculous meningitis is the most severe clinical form of disease caused by *M. tuberculosis* [4, 8]. It represents approximately 1 % of all TB cases, but is disproportionately important because it results in unfavorable outcomes including death and sequelae in one-third of the patients treated with antituberculous medications [9–11]. Therefore, early initiation of effective anti-TB therapy is essential for the success of TBM treatment [5]. However, in accordance with increasing drug resistance seen in TB cases worldwide, the number of drug-resistant TBM cases has been on the incline, making drug-resistant TBM a more challenging clinical problem [2, 8, 11].

Our results showed that, although 85.9 % of TBM cases were susceptible to four first-line drugs tested, 14.1 % were resistant to at least one anti-TB drug. According to the joint annual report by ECDC (European Centre for Disease Prevention and Control) and the WHO regional office for Europe, tuberculosis surveillance and monitoring in Europe 2014, the prevalence of resistance to at least one anti-TB drug in all TB cases is 12.7 % across European Union (EU) countries [12]. Given that the isolates tested in our study were obtained basically from European countries, it can be expected that the rate of susceptibility to all the four first-line drugs in our TBM cases would likely to be similar to that reported for all TB cases. This finding suggests that, in cases diagnosed with TBM, prompt initiation of standard empiric anti-TB treatment without awaiting the results of DST would not cause any problems in terms of drug resistance in about 85 % of cases. The rate of resistance to at least one drug in TBM cases was reported as 17.8 % from India, similar to that of our finding [7]. Another study conducted in Vietnam reported this rate as high as 40 % and drew attention to the problem of drug-resistant TBM [13].

Among the first-line anti-TB agents, INH represents one of the most valuable drugs in the treatment of TBM, in that it has good CSF penetration and has early bactericidal activity. Therefore, INH resistance can be considered to affect the success of TBM treatment more than that of pulmonary TB [2, 5, 7]. Continuation phase of treatment containing three drugs as a daily regimen should be given in countries with high levels of INH resistance in new TB patients, and where INH drug susceptibility testing in new patients is not done [7]. In our study, overall and monoresistance rates for INH were 12 and 6.3 %, respectively. These rates were higher than those found for other first-line drugs tested. Monoresistance to INH has been reported to have the highest rate in TBM cases in other studies [7, 14]. INH resistance in pulmonary TB cases was found to be 10 % in the European Union countries, with a higher rate of 15 % in Turkey [12]. These data suggest that INH resistance rates across this region are similar for pulmonary and central nervous system TB.

Reported rates of INH-monoresistance range from 6 to 6.5 % in TBM cases in accordance with our findings. These cases have significantly higher mortality rates compared to those with susceptibility to all first-line drugs [5, 15]. A study reported that 35 % of patients with INH-monoresistance and 28 % of patients with susceptibility to all first-line drugs died during treatment [5]. The mortality rates in our cohort are 33.3 % in patients with INH-monoresistance, compared with 23.8 % in patients without resistance to any first-line drugs. However, this difference was not statistically significant probably due to the low number of cases with resistance in our cohort.

In some regions in the world, nearly one-third of all TB cases is MDR [2]. The rate of MDR-TB among all TB cases has been reported to be about 5 % in the European Union countries and in Turkey [12]. Studies that report MDR-TBM rates among TBM patients are limited in number, most of which come from areas with high TB prevalence [7, 16]. These rates range from 4.3 to 8.6 % for Vietnam and South Africa [13, 15, 16] and from 1.5 to 2.4 % for the United States and India [7, 17]. There were only five (3.5 %) cases of MDR-TBM in our study, three from Turkey and two from Serbia.

According to the 2014 ECDC report, mortality occurred in 7.4 % of all TB cases across the European Union, with a striking rise to 17.8 % in MDR-TB patients; these rates were reported as 3.3 and 8.0 % for Turkey, respectively [12]. Although mortality rates for TBM and MDR-TBM were not specified in this report, these two conditions would apparently present with higher rates due to more severe disease characteristics [11, 16]. In preantibiotic era, the mortality rate in TB was 58 % [18]. In a recent meta-analysis, the average mortality rate in adult TBM cases in Africa was calculated as 60 % [19]. Several studies exist reporting that MDR is one of the most important prognostic factors for TBM, leading to significantly shorter survival [2, 8, 15]. In a study involving 180 adults with TBM, overall mortality rate was reported as 33.3 %, where all the patients with MDR-TBM (n = 10) died before completing the treatment (relative risk of death, 11.63; 95 % CI, 5.21–26.32) [13]. Another study reported mortality rates as 73 and 29 % among TBM patients with and without MDR, respectively, where MDR existence presented a relative risk of death of 2.49 (95 % CI, 1.95–3.18) [17]. In our study, the mortality rate of patients with MDR-TBM was higher (40.0 %) than that of cases with susceptibility to all drugs (23.8 %), and the relative risk of death for MDR-TBM was 2.14 (95 % CI, 0:34–13:42). As in monoresistance issue we could not display a statistically significant association for MDR isolates and mortality probably due to low numbers.

A meta-analysis of studies conducted in Africa reported the prevalence of HIV positivity in a range of 55 to 88 % among TBM patients [19]. HIV epidemics complicated by poor TB control programs resulted in increased number of MDR-TB cases, thus causing more and more patients to develop MDR-TBM in the past. The treatment of MDR-TBM is more challenging in HIV-positive patients, with a fatal course in more than 60 % of the cases [7, 11, 15, 20]. A striking difference was reported between the mortality rates of HIV-infected patients with and without MDR-TBM (88 % vs. 49 %). In addition, HIV infection can be considered a risk factor for MDR-TBM [17, 21]. The majority of studies on patients with coexistent HIV and MDR-TBM come from areas where both conditions manifest high prevalences. A study from Vietnam found HIV-positivity rates as 22.2 and 45.5 % in patients with TBM in absence of MDR and MDR-TBM, respectively, with insignificant associations between the patients with HIV infection and MDR-TBM [13]. In contrast, another study from the United States reported the coexistence of HIV infection in 89 % of MDR-TBM patients and found a strong correlation between HIV infection and MDR [17]. According to the 2014 ECDC report, although 5.5 % of all TB cases in the European Union countries had HIV infection, this rate was 0.5 % in Turkey [12]. There were only three HIV-positive patients (2.3 %) and all of MDR-TBM cases are HIV-negative in our study, because of the imbalanced recruitment of patients mainly from Turkey. It was not possible to assess the effect of HIV infection on the development of MDR-TBM and on mortality due to the small number of patients with positive HIV status. Consequently, comorbid conditions were not shown to affect TBM deaths in this study.

In MDR-TB cases, resistance to other anti-TB drugs other than INH and RIF has also been frequently reported [17]. In our study, two out of five MDR-TBM cases exhibited resistance to EMB, and the other two to both EMB and SM. In general, rifampicin resistance is considered to be a good marker to detect MDR-TB [22, 23]. This was consistent with our finding, in that all patients with resistance to RIF are MDR-TBM cases. Literature data supported by our finding suggest that utilization of molecular tests for the detection of RIF resistance alone may prove to be helpful in the rapid diagnosis of MDR-TBM [22]. However, it should be kept in mind that this can’t detect INH-monoresistance in which the treatment will be adversely affected.

Monoresistance to EMB is rare and there was no evidence of the negative effect of monoresistance to SM on TBM treatment [15]. Among our cases, only three patients exhibited monoresistance to EMB (n = 1) or SM (n = 2), and one of this patients with SM-monoresistance dead during treatment.

One limitation to the study is that participating centers used different DST methods owing to the retrospective nature of the study. However, it would very difficult to provide such a large TBM cohort prospectively. The numbers of INH monoresistant and MDR-TBM cases were low in our study and because of this, it lacked statistical power at this point.

## Conclusion

In conclusion, based on our findings, it can be inferred that INH-resistance and MDR seem not to be worrisome in TBM cases in our study. However, considering their adverse effects on treatment, early and prompt detection of at least INH- and RIF-resistance would be most beneficial for designing anti-TB therapy. Still, empiric TBM treatment should be started immediately while DST results are pending.
